# Beyond the Physical: The Mental Health Toll of Delayed Blood Work in Global Health Disparities

**DOI:** 10.3389/ijph.2025.1608382

**Published:** 2025-05-19

**Authors:** Haewon Byeon

**Affiliations:** Department of Future Technology, Worker’s Care and Digital Health Lab, Korea University of Technology and Education, Cheonan, Republic of Korea

**Keywords:** delayed blood work, mental health implications, healthcare disparities, psychological distress commentary, mental wellbeing

This article by Chung et al. [1], Delays in Blood Work and Disease Burden: A Cross-Sectional Analysis of Unmet Blood Work Need and Seven Key Health Conditions Across 21 Countries, presents a critical examination of disparities in access to essential healthcare services. As a public health scholar, I am deeply concerned by the study’s findings, particularly the significant mental health implications arising from delays in accessing basic medical procedures, such as blood work. While the authors meticulously document the prevalence of these delays and their devastating impact on physical health, the profound psychological toll on individuals remains largely unexplored.

The study reveals alarming disparities in access to blood work, with a staggering percentage of respondents in some countries never having undergone these crucial tests [1]. This disparity is most pronounced in regions burdened by significant disease prevalence, a deeply concerning observation. From a mental health perspective, the anxiety and uncertainty engendered by the inability to access timely diagnostic information cannot be underestimated [2]. The constant worry about one’s health status can significantly erode mental wellbeing, leading to increased anxiety and even depression.

Furthermore, the study highlights the vulnerability of individuals with existing medical conditions, who are disproportionately affected by delays in blood work. For those living with chronic illnesses, these delays can exacerbate existing psychological distress [3]. The fear of the unknown, the uncertainty surrounding their health trajectory, and the potential for missed opportunities for early intervention can all contribute to a significant decline in mental wellbeing. Moreover, delayed diagnoses can lead to more severe health complications, increasing the overall burden on individuals and their families, both financially and emotionally.

The authors rightly emphasize the critical role of socioeconomic factors in determining access to healthcare. Individuals with lower socioeconomic status, characterized by limited education and financial insecurity, are disproportionately impacted by these delays. This perpetuates a cycle of healthcare disadvantage, where individuals from marginalized communities face systemic barriers to accessing essential care. These experiences can profoundly erode an individual’s sense of control and self-efficacy, both crucial determinants of mental wellbeing. Addressing these systemic inequities in healthcare delivery is paramount for improving the mental health outcomes of vulnerable populations.

While this study primarily focuses on the physical health consequences of delayed blood work, its implications for mental health are undeniable. The conditions investigated, including malaria, HIV, and thalassemia, often carry significant social stigma and require ongoing medical monitoring. These factors can have a profound impact on an individual’s mental health, contributing to feelings of isolation, shame, and despair. Moreover, poor mental health can significantly impede adherence to treatment regimens and the ability to navigate complex healthcare systems, further exacerbating the cycle of health inequity.

Individuals experiencing these delays may be at increased risk for developing mental health conditions such as depression, anxiety, and even suicidal ideation [4]. The hopelessness and despair that can arise from a lack of access to timely care can have devastating consequences for mental wellbeing. It is crucial to recognize that the true impact of delayed healthcare services may be underestimated if we fail to adequately consider the psychological consequences.

From a clinical perspective, this study underscores the urgent need for a more integrated approach to healthcare that addresses both physical and mental health needs. Healthcare providers must be equipped to identify and address mental health concerns within their routine clinical practice. This may involve incorporating brief mental health assessments into routine physical health check-ups and developing readily accessible evidence-based psychological interventions within healthcare settings.

This study also has significant implications for public health policy. Addressing socioeconomic barriers to healthcare, reducing disparities based on factors such as education and income, and increasing access to care for all individuals are critical public health priorities. Widespread public health campaigns aimed at educating the public about the importance of routine blood testing and advocating for increased resources to ensure timely access to care for all are essential.

Future research should delve deeper into the specific mental health consequences of delayed blood work. Studies incorporating comprehensive mental health assessments alongside evaluations of physical health outcomes are crucial. Longitudinal studies are needed to investigate the long-term trajectory of these conditions and to evaluate the effectiveness of proposed public health interventions. Furthermore, understanding how cultural and environmental factors influence access to healthcare services and shape individuals’ perceptions of healthcare is essential for developing effective and culturally sensitive intervention strategies.

In conclusion, Chung et al.’s research provides a compelling argument for the urgent need to address the significant disparities in access to routine blood testing. While the physical health consequences of these delays are well-documented, the profound impact on mental wellbeing cannot be ignored. A truly person-centered approach to healthcare must prioritize the integration of physical and mental health services, addressing the complex interplay between these two critical dimensions of human wellbeing. By acknowledging and addressing the profound impact of health inequities on our collective wellbeing, we can strive to create a more just and equitable healthcare system for all.
